# Mycobacterial genomics and structural bioinformatics: opportunities and challenges in drug discovery

**DOI:** 10.1080/22221751.2018.1561158

**Published:** 2019-01-16

**Authors:** Vaishali P. Waman, Sundeep Chaitanya Vedithi, Sherine E. Thomas, Bridget P. Bannerman, Asma Munir, Marcin J. Skwark, Sony Malhotra, Tom L. Blundell

**Affiliations:** a Department of Biochemistry, University of Cambridge, Cambridge, UK; b Institute of Structural and Molecular Biology, Department of Biological Sciences, Birkbeck College, University of London, London, UK

**Keywords:** Mycobacterium, comparative genomics, structure-guided drug discovery, drug resistance, mutation

## Abstract

Of the more than 190 distinct species of *Mycobacterium* genus, many are economically and clinically important pathogens of humans or animals. Among those mycobacteria that infect humans, three species namely *Mycobacterium tuberculosis* (causative agent of tuberculosis), *Mycobacterium leprae* (causative agent of leprosy) and *Mycobacterium abscessus* (causative agent of chronic pulmonary infections) pose concern to global public health. Although antibiotics have been successfully developed to combat each of these, the emergence of drug-resistant strains is an increasing challenge for treatment and drug discovery. Here we describe the impact of the rapid expansion of genome sequencing and genome/pathway annotations that have greatly improved the progress of structure-guided drug discovery. We focus on the applications of comparative genomics, metabolomics, evolutionary bioinformatics and structural proteomics to identify potential drug targets. The opportunities and challenges for the design of drugs for *M. tuberculosis*, *M. leprae* and *M. abscessus* to combat resistance are discussed.

## Mycobacteria: the global disease burden

Mycobacteria belong to the genus *Mycobacterium*, which is the only genus representing the *Mycobacteriaceae* family (order: *Actinomycetales*; class: *Actinobacteria*). The genus was proposed in 1896, to include two species namely tubercle bacillus (now known as *Mycobacterium tuberculosis*) and leprosy bacillus (*Mycobacterium leprae*) [1]. Currently, there are >190 distinct species of *Mycobacterium* genus, some of which are economically and clinically important pathogens of humans or animals [2].

Among the human mycobacterial infections, those caused by *M. tuberculosis* (tuberculosis), *M. leprae* (leprosy) and *Mycobacterium abscessus* (chronic pulmonary infections) pose a public health concern. Mycobacterial infections affect ∼11–14 million people each year globally and tuberculosis (TB) alone is responsible for ∼1.3 million deaths each year, of which 374,000 were people living with HIV/AIDS. In 2016, 10.4 million people living with HIV/AIDS, were diagnosed with TB [3].

In case of *M. leprae* infections, the World Health Organization (WHO) reported ∼200,000 new cases of leprosy each year (http://www.who.int/wer/2017/wer9235/en/). In 2016, 214,783 new cases of leprosy were reported globally. India reported the highest number (135,485) and Brazil 25,218 cases. Lack of effective early diagnostic tool(s), vaccines and limited understanding of the patterns of transmission contribute to the ongoing incidence.

In addition to TB and leprosy, Non-Tuberculous Mycobacteria (NTM), such as *M. abscessus complex,* are emerging worldwide as the cause of chronic pulmonary infections [4,5]. Incidence rates of pulmonary infections due to NTM, vary greatly with geographical regions. The global incidence remained 1.0–1.8 in a population of 100,000, however, these rates are much higher in the US, Western Pacific and Canada [5]. The prevalence of NTMs has increased from 1.3% [6] to 32.7% in Colorado, in cystic fibrosis patients [7].

### The growing challenge of antimicrobial resistance

The past few decades have seen a “discovery void” pertaining to antibacterial drug development, where very few new molecules have been patented or approved for clinical use [8]. This is particularly true for drug discovery against mycobacteria, where no new drugs that were specifically developed for this purpose reached the clinic after the early 1960s until recently [9]. Importantly, the emergence of resistance towards first-line and second-line drugs poses additional challenges to the development of suitable drugs in each of *M. tuberculosis*, *M. leprae* and *M. abscessus*. The approval of drugs and causes of resistance have been systematically reviewed for *M. tuberculosis* [10,11], *M. abscessus* [12] and *M. leprae* [13]. Antibiotic resistance can arise due to physiological, intrinsic or acquired factors [10,14]. The intrinsic resistance is attributed to cell-wall permeability, drug efflux systems, drug targets with low affinity and enzymes that neutralize drugs in the cytoplasm. The acquired resistance is conferred by chromosomal mutations.

Although the TB mortality rate is falling at ∼3% per year globally, drug-resistant TB remains a continuing threat [3]. The spread of drug-resistant strains of TB (mono-resistant, multidrug-resistant, extensively drug-resistant and totally drug-resistant) is alarmingly high and accounted for 490,000 cases of multidrug-resistant TB (MDR-TB) in 2016. Around 47% of the MDR-TB cases are reported in Southeast Asia [15]. Increase in isoniazid-susceptible rifampin resistance was also noted in 2016, with 110,000 cases globally. The global burden of MDR-TB has recently increased by >20% annually [16] and the treatment is successful in only 50% of the MDR-TB cases. With the ongoing transmission of the drug-resistant strains of *M. tuberculosis* in communities, it is increasingly important to research novel drug targets and identify potential leads that can be expanded to new drugs.

Drug resistance in leprosy is diagnosed by the mouse footpad method, which is time and labour-intensive. Alternatively, mutations can be detected in drug-resistance-determining regions of *M. leprae* drug targets such as dihydropteroate synthase (for the drug: dapsone), β subunit of RNA polymerase (rifampin) and subunit A of DNA gyrase (ofloxacin). As drug resistance has been suspected only in cases that self-report at the hospital as a result of reactivation or relapse in leprosy, the numbers are currently low; however, these may increase if a field-based surveillance system is implemented.

*M. abscessus* is naturally resistant to many first-line antimicrobials, including all the current TB drugs. The acquired resistance to aminoglycosides is known to be due to mutations in genes such as *rrs* [12]. Likewise, mutations in 23S rRNA and genes such as *erm(41)* and *rrl* are known to cause macrolide resistance in *M. abscessus* [17].

**Figure 1.**
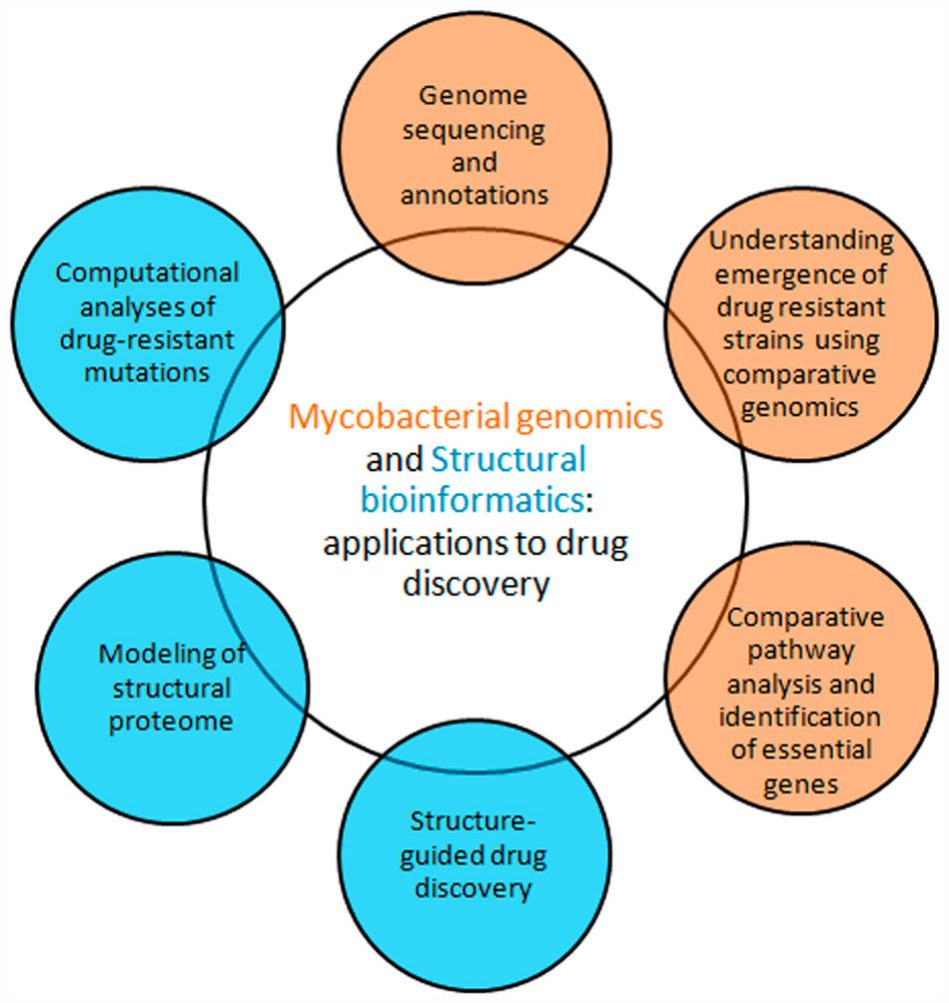
From mycobacterial genomes to drug discovery. Post-genomic application areas in mycobacterial drug discovery such as comparative genomics and structural biology/bioinformatics are shown.

Thus, emergence of antibiotic-resistant strains and rapid spread due to globalization poses a serious challenge to the global health [18]. Genomics has served as an important milestone in bacterial drug discovery [19,20]. It is now possible to understand causes of emergence of antibiotic-resistant strains and to identify potential drug targets through combinatorial approaches involving comparative genomics, metabolomics, phylogenomics, evolutionary and structural biology/bioinformatics (Figure 1). Here we discuss the current status of drug discovery research in each area, focusing on the scope and applicability of computational approaches.

## Availability of genome sequences and annotation data: applications of comparative genomic approaches to drug discovery research

Comprehensive understanding of the organization of mycobacterial genomes began in 1998 with the elucidation of the complete genome sequence of *M. tuberculosis* [21,22]. The *M. tuberculosis* reference genome (H37Rv strain) is 4.41 Mbp in length and comprises 4081 protein genes, 13 pseudogenes, 45 tRNA genes, 30 ncRNA, 3 rRNA genes and 2 miscRNA genes (http://svitsrv8.epfl.ch/tuberculist/).

The genome of *M. leprae* was first sequenced in 2001 [23]. This 3.2 Mbp genome has significant sequence similarity with that of *M. tuberculosis*; however, *M. leprae* reductively evolved to survive with 1614 protein genes, 1310 pseudogenes, 45 tRNA genes, 3 rRNA genes and 2 stable RNA genes (http://svitsrv8.epfl.ch/mycobrowser/leprosy.html).

The complete genome of *M. abscessus* strain ATCC 19977 was first sequenced in 2008 [24]. The 5.06 Mbp genome consists of 4941 genes encoding 2886 proteins with functional assignments and 2055 hypothetical proteins (https://www.patricbrc.org/view/Genome/36809.5).

Rapid annotation of genomic data, leading to the development of general purpose as well as specialized resources, described in Supplementary Table 1, is providing important information pertinent to sequence, structure, function, metabolic pathway, taxonomy and drug resistance mutations.

### Comparative genomics: understanding strain diversity and emergence of drug-resistant strains

Most pathogenic mycobacterium species including *M. tuberculosis* and *M. leprae* are slow growers taking >7 days to form visible colonies on solid media. Comparative pan-genomic analyses indicate that the evolution of rapid and slow growers is attributed to a series of gene gain and gene loss events leading to adaptation to different environments [25]. Classical methods for genotyping of mycobacterial strains include IS6110DNA fingerprinting, spoligotyping and 24 locus-MIRU (mycobacterial-interspersed repetitive units)-VNTR (variable number of tandem repeats) typing [26]. With the availability of genome sequencing data, complete genome-based phylogenies are being used for genotyping and classification of mycobacteria [27,28]. The classical *M. tuberculosis* complex is subdivided into seven distinct lineages with characteristic geographic distribution [29]. Genomic analyses revealed three subspecies of *M. abscessus*, namely *M*. *abscessus*, *M. massiliense* and *M. bolleti* [30]. Likewise, lineage diversity within distinct subtypes of *M. leprae* has been recently studied based on phylogenomic analyses of 154 genomes [31].

Molecular phylogenetics and evolutionary dynamics methods allow study of epidemiology and links between genetic diversity and emergence of drug resistance in mycobacterial strains [32]. Antibiotic-resistant genes are important markers for delineation of evolution and spread of drug resistance [10,14,33]. Such computational studies have helped to answer specific questions such as: Whether a particular phylogenetic lineage is associated with drug resistance in an outbreak/epidemic? When and how the drug-resistant strains have emerged? What are the evolutionary factors that cause antimicrobial drug resistance in mycobacterium? Computational studies on mycobacterium addressing each of these questions are described below.

Recently, genomic studies focusing on population structure, origin and spread of MDR-TB have been undertaken across various parts of the world [29,34–36]. A study [34] on strain diversity and phylogeography using genomes of 340 *M. tuberculosis* strains (isolated during 2008–2013) from KwaZulu-Natal, helped to elucidate the timing of acquisitions of drug resistance mutations that confer XDR-TB, indicating that isoniazid-resistance evolved earlier than rifampicin-resistance^.^ Similarly, molecular epidemiology of MDR-TB in Ireland has been systematically examined using genomes of *M. tuberculosis* strains isolated during 2001–2014 from MDR-TB cases [35]. Among seven lineages of MTB complex, Beijing lineage was observed to be associated with MDR [35]. MDR-TB in Ireland was found to be introduced from other localities, as known for several European countries [37].

In the case of *M. abscessus*, phylogenomic studies revealed the major role of recombination in causing lineage diversity [30,38,39]. Population structure and recombination analyses provided significant evidence of gene flow and admixture among three lineages (*M. abscessus*, *M. massiliense*, and *M. bolleti*), and a correlation with pathogenicity and macrolide resistance in cystic fibrosis patients was found [39]. Phylogenomic and genetic polymorphism analyses have also been carried out using *M. abscessus* isolates from US [40]. A population genomic study [41], based on the worldwide collection of clinical isolates of *M. abscessus,* has shown that the majority of *M. abscessus* infections are acquired through transmission (potentially via aerosols and fomites) of recently emerged circulating clones that have spread across the world. These clones are observed to be associated with increased virulence and worse clinical outcomes. This is a wake-up alarm! We are facing a pressing international infection challenge [41].

In the case of *M. leprae*, a comprehensive study on phylogenomics and antibiotic resistance has been recently published [31], which focuses on sequence and selection pressure analysis of wildtype as well as antibiotic-resistant genes. Several attempts have also been made to analyse the origin and spread of leprosy across various parts of the world [42].

Thus, comparative genomics and phylogeographic studies are important in understanding the global spread and transmission dynamics of the mycobacterial infections. Strain diversity is shown to be one of the contributing factors to antibiotic resistance, linking transmission dynamics to medicine, across various geographic areas which are endemic for diseases such as TB [43]. Furthermore, all these developments point to an increasing need for new and effective drugs to combat antibiotic resistance. Ideally, new drugs must have a novel mode of action to reduce events of cross-resistance, optimal dose-response, pharmacokinetic and toxicity profiles allowing safe and short duration of therapy either solely or in combination with other drugs. Comparative genomics of metabolic pathways provide a means to rapidly identify potential gene targets and thus could aid in drug discovery research (detailed in next section).

### Comparative pathway analyses and identification of essential genes

*M. tuberculosis*, *M. abscessus* and *M. leprae* survive in distinct ecological niches with distinct evolving features that enable them to adapt to their specific environments. Malhotra et al. [44] describe a 90% overlap of known and proposed drug targets against all mycobacterial species. The study identified the major pathways such as chorismate, purine/pyrimidine and amino acid biosynthetic pathways, essential for the survival of mycobacteria, as conserved in all these three species with minor differences in the carbon metabolism pathways.

*M. tuberculosis* is known for its peculiar ability to survive in the human macrophage despite varying stress conditions (redox, acidic, and nitrosamine) within the host. This has contributed to a very elaborate DNA repair and recombination system. The most up-to-date metabolic reconstruction for *M. tuberculosis* describes 1228 metabolic reactions, 1011 genes and 998 metabolites [45], where 250 protein-coding genes present in the lipid biosynthetic pathways are responsible for the thick cell wall. Mutations in these pathways give rise to further changes in their metabolic pathways.

Draft metabolic reconstruction for *M. abscessus* exists in BioCyc [46] and KEGG [47] databases, with 110 pathways including biosynthesis, metabolism, biodegradation and information processing pathways. Evolutionary analyses showed that *M. abscessus* is more closely related to other NTMs such as *M. avium* complex and has a well-supported membrane transport system with a large number of efflux pumps, resulting in multidrug-resistant features [48]. As an opportunistic bacterium, *M. abscessus* is able to survive outside its host and also contains metabolic pathways not associated with pathogenesis, which contribute to its larger genome size as compared to obligate pathogens.

In case of *M. leprae*, reductive evolution has led to the loss of common catabolic pathways such as lipolysis and impairment of the energy metabolism pathways [23]. Moreover, the production of cognate and prosthetic groups from transport, biosynthetic and electron transfer pathways is also affected [49]. With the loss of several functions, and the presence of a large number of conserved but unknown functions of pseudogenes, further studies to characterize the metabolic pathways in *M. leprae* are required.

### Approaches to identify essential genes and prioritization of drug targets

The availability of genome sequences of mycobacteria, and annotations in sequence, structure and pathway databases facilitate systems-level analysis wherein comparative genomics and evolutionary bioinformatics approaches can be integrated to identify the minimal set of essential genes, leading to faster identification of putative drug targets. Essential genes that are necessary for the survival of the bacterium, and are critical components of metabolic and physico-chemical pathways, can be identified by gene knockouts, saturation transposon mutagenesis, RNA interference, etc. Essential genes have been characterized experimentally in the case of *M. tuberculosis* [50–52]. However, limited studies have been carried out in *M. abscessus* and *M. leprae*. Specifically, *M. leprae* cannot be cultured *in vitro* and thus demands computational identification of essential genes.

Computational approaches for identification of essential genes, including machine learning, flux balance analyses and comparative genomics [53], are faster and cheaper than experimental methods [53]. Machine learning methods utilize unique genomic features of essential genes such as length of proteins, codon usage, GC content, sub-cellular localization, higher rate of evolutionary conservation, etc. Dedicated resources such as Database of Essential Genes [54] and the database of Online Gene Essentiality [55] have been developed, which serve as a platform for identification and prioritization of essential drug targets in various species including *M. leprae* [56]. Advent of next-generation sequencing has enabled design of comparative genomics workflows to identify essential genes in case of *M. tuberculosis* [57].

Upon identification of a set of essential genes, prioritization of drug targets can be achieved by further screening of essential genes based on computational predictions of ADMET (absorption, distribution, metabolism, excretion and toxicity) properties, sub-cellular localization, etc. In view of growing concern about drug resistance, attempts have been made to prioritize drug targets based on various analyses such as identification of uniqueness in metabolome and similarities to known druggable proteins, analysis of the protein-protein interactome, flux balance analysis of reactome and sequence-structural analysis of targetability of genes using integrative strategies [58,59]. As most critical targets in a pathogenic organism are expected to be evolutionarily conserved, this has been applied to filter essential genes in *M. tuberculosis* [58]. Likewise, convergent positive selection analyses were used to identify genes that possibly cause drug resistance in *M. tuberculosis* [59]. Evolutionary rate has been proposed as a useful parameter for ranking and prioritizing antibacterial drug targets [60]. Thus, understanding evolution of drug targets is one of the important aspects of the drug discovery, especially to tackle the problem of drug resistance.

## Structural biology and bioinformatics to combat mycobacterial infections

Early approaches to the discovery of new antibiotics relied almost entirely on whole-cell phenotypic screening of natural products, microbial extracts and fermentation broths. This approach has helped in the discovery of most antibiotics in use to date and recently led to the approval of two new drugs, bedaquiline and delamanid, for the treatment of drug-resistant strains of TB [61]. Structure-guided drug discovery, pioneered in academia and some companies in the 1970s became central to the discovery of antihypertensives that targeted renin and AIDS antivirals that targeted HIV protease in the 1980s and 1990s [62–64]. Opportunities for structure-guided drug discovery of mycobacterial targets became real only later when genome sequences became available. Understanding the structure and mechanism of the target allowed progress and optimization of hit-to-lead molecules, as well as a better understanding of resistance mechanisms, identifying causes of potential side effects and drug-drug interactions. This understanding led many research groups to perform high-throughput screening (HTS) and fragment-based screening campaigns of chemical libraries [65] directly against carefully selected targets of interest. The applications of structure-guided drug discovery to mycobacteria have been reviewed earlier [66–68].

Structural features can also be used to further refine target selection and validation including lack of structural homology to human host to avoid mechanism-based toxicity and ligandability leading to modulation of target activity. Where the target protein has not been structurally or functionally characterized, it’s structural model can be built based on homologous proteins using programs like MODELLER [69]. Important information regarding target protein function and properties can be obtained from databases such as TubercuList (http://svitsrv8.epfl.ch/tuberculist/) or CHOPIN [70]. Further, druggability of targets can be predicted by analysing properties and depths of various pockets/hotspots (capable of small-molecule binding interactions), using several databases and programs [71].

### Fragment-based drug discovery (FBDD): a promising alternative to HTS approach

**Figure 2.**
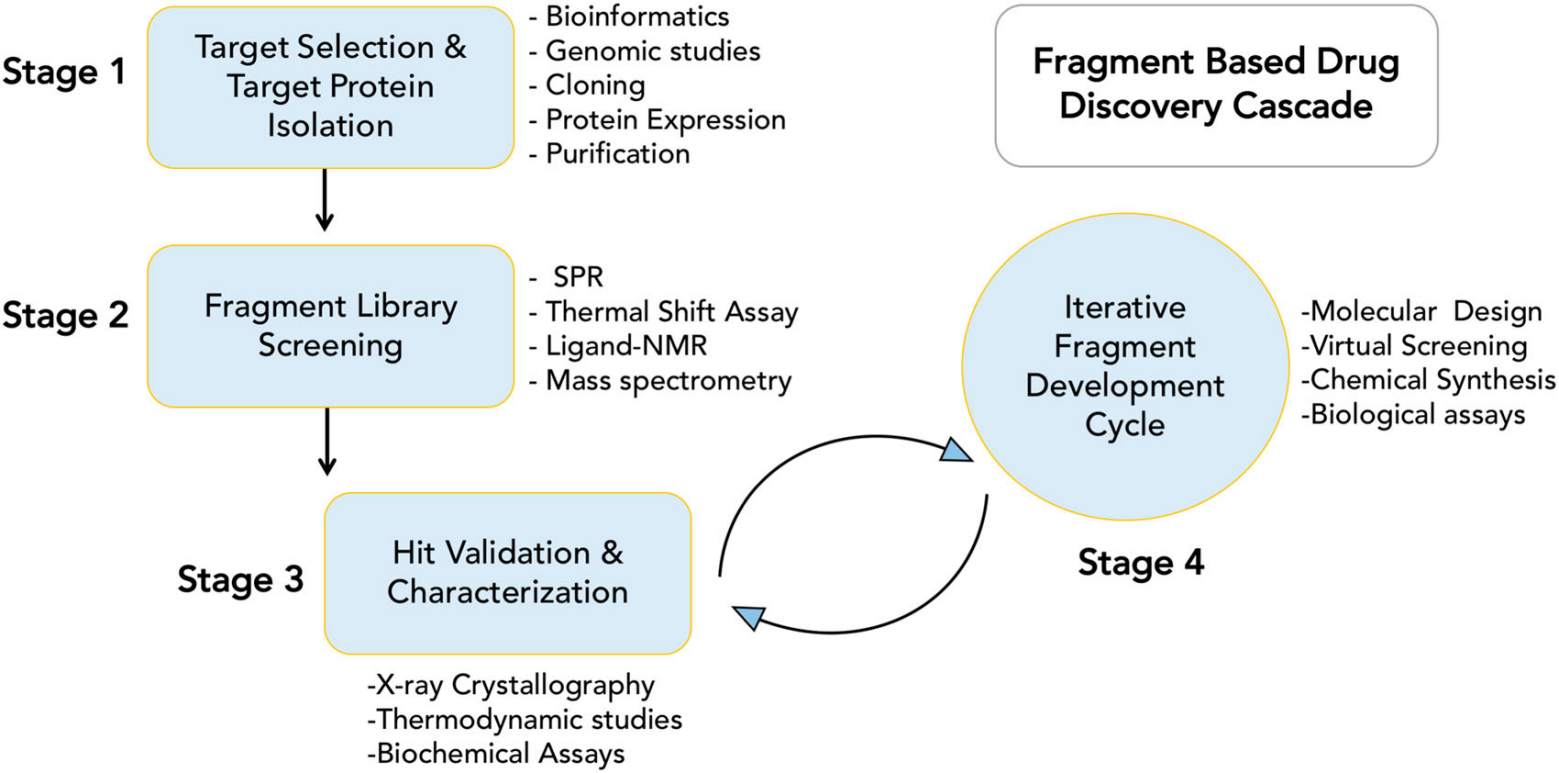
FBDD cascade. Various techniques involved in each of the four stages are shown.

The principal advantage of the FBDD approach compared to HTS methods is that very small libraries (<10^3^ fragments) of low molecular weight (<300 Da) compounds can be used to obtain good starting points for lead discovery [65]. Initial fragment hits usually exhibit lower potency than the more complex molecules found in typical HTS compound libraries, but can be chemically optimized into lead candidates, thereby more effectively exploring the chemical space available for binding to the target protein [68]. Figure 2 illustrates a typical fragment screening cascade employed in our lab. The details of these steps are summarized in Supplementary File 1.

FBDD approaches have been successfully applied to design inhibitors targeting various key *M. tuberculosis* enzymes such as pantothenate synthetase [72], transcriptional repressor [73], cytochrome P450 [74], thymidylate kinase [75] and malate synthase [76]. Some of the lead molecules developed from such fragment-based campaigns showed promising inhibitory response against *M. tuberculosis*, as shown in the example in Supplementary Figure 1.

### Virtual screening, hotspot mapping and pharmacophore modelling to aid structure-guided drug discovery

*In-silico* screening and docking of compound libraries often help to reduce the time and cost involved in experimental testing of large sets of compounds to identify potential hits. The effectiveness of such virtual screening exercises can be improved by complementing the analysis with fragment hotspot-mapping programs [71], which identify regions within the protein that provide relatively large contribution towards ligand binding in addition to information on interactions governing the predicted regions. Several studies [77,78] have used an energy-based pharmacophore modelling approach to complement virtual screening, followed by chemical optimization to identify inhibitors. We have collaborated with Maria Paola Costi at the University of Modena in a similar study involving a combination of virtual screening and molecular dynamics simulation methods to identify novel chemical scaffolds targeting *M. tuberculosis* thymidylate synthase X [79].

Phenotypic screening along with advances in chemical genetics and bioinformatics has allowed target-guided compound identification and optimization [80], by utilizing target mechanism-based whole-cell screening or by genetic manipulation of the target phenotype, or by finding targets directly from phenotypic hits [81]. The latter approach can employ various techniques such as whole genome sequencing to identify resistant mutants [82,83], transcriptional profiling [84], chemical biology and metabolomics [85] or in-silico methods [86]. These advances in the field discussed above, coupled with a comprehensive understanding of the chemical space of mycobacterial drugs, hold promise of accelerating the process of mycobacterial drug discovery.

### From genomes to proteomes: automated modelling of proteomes through structural genomics and bioinformatics

Methods to identify structures that are potentially similar to the protein in question (*templates*) have been developed in our laboratory [87] and elsewhere (reviewed in [88]), as well as methods such as MODELLER [69] and Rosetta [89] to leverage this similarity to build theoretical, comparative models based on target-template alignments. Community-wide efforts, such as Critical Assessment Structure Prediction [90] have led to substantial improvements in the accuracy of these approaches, both in terms of template identification and alignment, as well as model building and refinement. However, most of these methods focus on producing a single, most likely structure of each protein.

Our group has developed a structural genomics resource for *M. tuberculosis* (H37Rv strain) called CHOPIN [70] which provides an ensemble of predicted models in different conformational states. These models are generated through a homology modelling pipeline. Development of such structural resources is important to identify potential therapeutic targets [91].

### Moving beyond proteomes: understanding the impact of drug-resistant mutations in drug targets

Mutations are likely to confer drug resistance by altering the energy landscape of the target protein, affecting the protein-protein interactions or affecting the drug/ligand binding with the target protein. Computational approaches to predict the effects of mutations on the structure and function of proteins can prove helpful in understanding the mechanism of drug resistance. Our lab has developed two well-established methods, SDM (Site Directed Mutator), based on a statistical approach using Environment Specific Substitution Tables [92] and mCSM (mutation Cutoff Scanning Matrix), a machine learning approach [93] to predict the structural and functional effects of mutations on the target proteins. mCSM is available in different flavours to predict the effects of mutations on protein stability (mCSM-stability), protein-protein interactions (mCSM-PPI) and protein-ligand interactions (mCSM-lig) [94]. Additionally, in order to determine the impact of mutations on flexible protein conformations, tools like EnCOM [95] and FoldX [96] have been developed. Such methods have been used by our group and others to gain insights into the mechanism of mutations in various genetic and mycobacterial diseases including leprosy [97–100]. Such analyses of the structural and functional mechanism of drug resistance causing mutations using computational approaches are very helpful in the rapid assessment of many mutations not easily achieved using experimental methods.

## Challenges and future perspectives

This review has focused on the importance of advances in sequence and structure determination of genomes and their protein products in efforts to develop structure-guided drug discovery against three mycobacteria: *M. tuberculosis*, *M. abscessus* and *M. leprae*. Although *M. tuberculosis* is the most studied mycobacterium, 3D structures of only ∼16% of gene products have been determined experimentally. Sequence-structure homology recognition and comparative modelling have proved useful in providing clues about druggability. Furthermore, the more accurate models, based on structures of close homologues, can provide a basis for virtual screening and ligand design.

Metabolic reconstructions are providing insights into how *M*. *tuberculosis* has adapted within the host, and similar studies in *M. abscessus* and *M. leprae* are needed to provide better understanding of pathogenesis and design of new treatment regimes. The current focus in many laboratories is on providing user-friendly databases for the structural proteomes, and extending these not only to structures of homo- and hetero-oligomers, and complexes with natural and synthetic ligands, but also to incorporate data on metabolic pathways and epistasis, which are important in target selection.

The target-guided approach for antibiotic drug development has generally led to a low rate of translation of *in-vitro* inhibitory response to bactericidal/bacteriostatic activity. This challenge requires further study not only of physico-chemical properties of compounds affecting permeability of the thick waxy cell envelopes of mycobacteria, but also of drug efflux pumps and metabolic inactivation of compounds by bacterial/host cell enzymes that affect compound bioavailability. The emergence of drug resistance through mutations in the target is also a growing challenge. We have developed systematic mutagenesis studies using our software with a view to identify regions where drug design might be directed with a minimal chance of resistance. This is likely to be a key component in drug discovery in future, especially through structure-guided fragment-based approaches, where choices of fragment elaboration or cross-linking can be made in the light of such analyses of mutability.
